# Lipopolysaccharide induced mouse depression model can better simulate changes in peripheral blood FFAs in alMDD

**DOI:** 10.1371/journal.pone.0340967

**Published:** 2026-01-23

**Authors:** Jincai Pan, Guanxi Liu, Yuyuan Wang, Yuting Lan, Yanmei Liang, Weicheng Li, Kexin Zhu, Yanling Zhou, Yuping Ning

**Affiliations:** 1 The First School of Clinical Medicine, Southern Medical University, Guangzhou, China; 2 The Affiliated Brain Hospital, Guangzhou Medical University, Guangzhou, China; 3 Key Laboratory of Neurogenetics and Channelopathies of Guangdong Province and the Ministry of Education of China, Guangzhou Medical University, Guangzhou, China; 4 Guangdong Engineering Technology Research Center for Translational Medicine of Mental Disorders, Guangzhou, China; West Bengal State University, INDIA

## Abstract

Numerous studies have highlighted a strong association between free fatty acids (FFAs) and major depressive disorder (MDD), however, the underlying mechanisms remain poorly understood. Here, we demonstrate that peripheral blood FFAs levels are significantly reduced in adolescent patients with MDD (alMDD), as quantified by gas chromatography–mass spectrometry (GC-MS), providing critical insights for preclinical depression research. Both chronic social defeat stress (CSDS) and lipopolysaccharide (LPS) models induced depression-like behaviors and triggered varying degrees of variant expressions in peripheral blood FFAs in mice. Specifically, the LPS-induced model generally exhibited superior fidelity in recapitulating the items of FFAs profile of MDD patients. Notably, hexanoic acid (C6:0), nonanoic acid (C9:0) and nonadecylic acid (C19:0) levels in CSDS mice showed closer alignment with those in alMDD individuals, whereas, cis-9-palmitoleic acid (C16:1), and myristic acid (C14:0) levels in LPS mice more accurately simulated the FFAs alterations observed in alMDD. Collectively, this study identifies the LPS-induced depression-like model as a more reliable proxy for investigating FFAs dysregulation in alMDD.

## Introduction

Major depressive disorder (MDD) is a worldwide mental disorder seriously affecting psychosocial functions with a bad quality of life, accounting for the biggest share of the world’s burden of disease in the top ten causes disability [1]. It is reported that the lifetime/life-span risk of recurrence is 60% after the first depressive episode and 90% after the third depressive episode [2]. With no conclusive clinicopathologic features, 30–40% of individuals with MDD do not respond to the antidepressants, leaving treatment insufficient to adequately reduce the suicidal ideation and behavior [3–5]. Notably, compared to adult patients MDD, younger MDD populations exhibit more pronounced social dysfunction and functional impairment, along with substantially elevated suicide risk – the leading cause of mortality among youth aged 15–19 years globally [6]. Studies show that adolescents with major depressive disorder (alMDD) exhibit a higher risk of onset, more severe symptoms, greater prevalence of psychotic features, and increased suicidal tendencies compared to adults with MDD [7,8]. alMDD exhibit lower therapeutic efficacy compared to adults, particularly when treated with a combined regimen of fluoxetine and cognitive behavioral therapy (CBT), which demonstrates significantly elevated remission rates [9]. Given the heightened neuroplasticity and distinct psychosocial stressors in adolescence, targeted investigation of MDD within this developmental window is essential to unraveling etiological pathways. However, current evidences on pathogensis in alMDD individuals remain unclear, therefore, identifying targets to elucidate the pathogensis in alMDD is an urgent priority.

The occurrence of alMDD is generally regarded as the interactions of genotype and environment. The metabolites and metabolic procedure play an important role anchoring such interaction in MDD [10,11]. Metabolite alteration occurs earlier in the pathogenesis and development of MDD, providing sensitive feedback on its characteristics of MDD, which contributes the effective signals to prevention, diagnose and therapy to MDD [12]. Studies are growing for most antidepressants focusing on multiple pathways involving extensive metabolic alterations in energy and lipid metabolism [13,14]. The role of lipid metabolites has been implicated in MDD and some hypothesis-driven studies of MDD [15–17]. Abnormal lipid metabolism frequently exists in individuals with MDD [18]. Nevertheless, the majority of prior metabolomics investigations in MDD have predominantly focused on adult populations. Evidence suggests that the neurobiological underpinnings of developmental-stage alMDD exhibit distinct pathophysiological signatures compared to adult-onset MDD, potentially explaining the diminished therapeutic efficacy of conventional antidepressants in alMDD cohorts [19]. Paucity of evidences add the obstacle to parse the pathogenesis of alMDD in lipid profiling. Accordingly, more researches are needed to address the lipid metabolites for getting further understanding on alMDD.

Free fatty acids (FFAs) are important lipid components of metabolites, gene-transcript abundance within the fatty acid desaturase FADS cluster demonstrated a causal association with alMDD [16]. As fundamental components of membrane phospholipids, FFAs play a significant role in physiology function and pathological mechanism. Researches had indicated that FFAs could serve as second messengers or modulators of complex signaling networks unique to mammals, functioning as “lipokines” [16,17]. Lipokines have been linked to various pathological conditions, including arthritis, coronary artery disease, inflammatory bowel disease, asthma and MDD [20–22]. Additionally, FFAs have been recognized as significant factors in MDD. For example, omega-3 polyunsaturated fatty acids, a subtype of FFAs, have been supported by plenty of studies as effective in improving MDD symptoms [23,24]. Furthermore, previous research suggests that FFAs hold promise as a diagnostic tool for identifying first-episode alMDD [25]. However, the majority of studies examining the association between FFAs and alMDD have predominantly emphasized observational clinical correlations, mechanistic inquiry into causal linkages and their underlying pathophysiological pathways remain critically underexplored. Therefore, further investigation through animal models is warranted to elucidate the specific mechanisms by which FFAs contribute to depression. However, it remains unclear which animal models can best replicate the alterations in peripheral blood FFAs observed in alMDD.

Chronic social defeat stress (CSDS) is a widely accepted model in understanding the molecular mechanisms underlying affective-like disorders by pressing prolonged social stress on mice [26]. Lipopolysaccharide (LPS)-induced depression-like model was also a method to construct depression-like behavior which was related neuron inflamation [27]. Both models are essential methods in preclinical study of alMDD. Employing Gas Chromatography–Mass Spectrometry (GC-MS) to assess the translational relevance of these preclinical models in recapitulating FFAs dysregulation observed in alMDD, our study quantitatively profiled the peripheral blood for FFAs in alMDD, healthy controls (HC), CSDS mice and LPS mice. Our finding indicates that LPS-induced depression-like behavior mice could produce FFAs concentration fluctuation more closely resembling those observed in alMDD.

## Methods

### Study design and data sourses

The original study involving human participants was approved by Institutional Review Board (IRB) of The Affiliated Brain Hospital of Guangzhou Medical University (Number: AF/SC-08/02.3) and was conducted according to the principles expressed in the Declaration of Helsink. All participants and legal guardians provided written informed consent following detailed protocol disclosure, along with rigorous quality control. All alMDD were recruited from the Affiliated Brain Hospital of Guangzhou Medical University from 1 February 2021–1 January 2023. Concurrently, population-based matched healthy controls were recruited through advertisement at the same hospital and in the Guangzhou community.

First episode alMDD were recruited from a parent randomized, double-blind, sham-controlled trial investigating intermittent theta-burst stimulation (iTBS) efficacy in adolescent and young adult MDD (AYA-MDD; ClinicalTrials.gov ID: ChiCTR2100042346), conducted in full compliance with Consolidated Standards of Reporting Trials (CONSORT) guidelines.

The diagnosis of alMDD was confirmed by board-certified psychiatrists using the Diagnostic and Statistical Manual of Mental Disorders, 5th edition (DSM-5) criteria. Inclusion criteria required participants to: (1) Be aged 12–18 years; (2) Meet alMDD diagnostic thresholds on the Structured Clinical Interview for DSM-5 (SCID-5); (3) Score ≥17 on the 17-item Hamilton Depression Rating Scale (HAMD-17); (4) Maintain psychotropic medication-naïve status for ≥4 weeks prior to screening; (5) Demonstrate protocol compliance through completion of ≥90% baseline assessments; and (6) Provide written informed assent with parental/guardian consent. Individuals were excluded if they met any of the following criteria: (1) Current alcohol/substance use disorders; (2) Major medical comorbidities (neurological, endocrine, autoimmune, or infectious diseases; traumatic brain injury; central nervous system pathology); (3) Lifetime history of seizure disorders; and (4) Pregnancy or lactation status. HC were required to have no lifetime psychiatric diagnoses or psychotropic medication exposure.

This retrospective study utilized original datasets accessed for research purposes on 13 May, 2024. All data were fully de-identified prior to analysis, with no access to personally identifiable information during or after data collection. A total of 204 participants were included in this study, comprising 88 healthy controls (HC) and 116 first-episode adolescent depression patients.

### Animal and behavioral test

#### Animal.

All procedures involving animals were approved by the Institutional Animal Care and Use Committee of Guangzhou Medical University (Acceptance Number: S2024-549) and conducted in accordance with guidelines of the National Institutes of Health (NIH) on the care and ethical treatment of animals. All animals were purchased from Guangdong Medical Laboratory Animal Center. Their maintenance and use were in accordance with protocols. After blood collection, the mice were immediately euthanized by cervical dislocation, and all efforts were made to minimize suffering. Mice had ad libitum access to food and water unless stated otherwise, and were housed four to five per cage in a temperature (23 ± 1°C) and humidity-controlled (40–60%) environment with a 14h light, 10h dark cycle. All experiments were performed on male mice (6–8 weeks old at the beginning of the experiments).

**Methods of sacrifice:** Animals were immediately euthanized by rapid decapitation following blood collection. This method severs the connection between the spinal cord and brainstem, ensuring immediate loss of consciousness and minimizing potential pain.

**Methods of anesthesia and/or analgesia:** No anesthesia or analgesics were administered. The rapid decapitation procedure (typically completed within seconds) is appropriate for small rodents (e.g., mice or rats) and is exempt from anesthesia requirements under specific conditions as per relevant Animal Welfare Guidelines.

**Efforts to alleviate suffering:** All procedures were performed by trained personnel to ensure swift and precise execution to minimize distress. The experimental design adhered strictly to the principles with efforts to shorten pre-euthanasia handling time and avoid unnecessary stress.

#### Chronic social defeat stress (CSDS).

Male adolescent C57BL/6J mice underwent daily 5-minute aggressive encounters with novel male CD-1 aggressors. Following each session, experimental mice were co-housed with their respective aggressors in perforated transparent partition-divided cages overnight, maintaining continuous sensory contact while preventing physical interaction. This protocol was repeated daily over 10 consecutive days, with experimental subjects exposed to a new aggressor each day. Control groups were maintained in identical housing configurations with daily rotation of non-aggressive conspecifics. All CSDS procedures were conducted during the light cycle.

#### Lipopolysaccharide (LPS).

Lipopolysaccharide (LPS; Sigma-Aldrich, MO, USA) was dissolved in 0.9% saline at a concentration of 0.05 mg/ml. Male adolescent C57BL/6J mice received daily intraperitoneal injections of LPS at a dose of 0.5 mg/kg/day for 7 consecutive days.

#### Force swim test (FST).

Individual mice were placed in a water-filled cylinder (20 cm diameter × 50 cm height) maintained at 23 ± 0.5°C. The 6-minute forced swim test was conducted under standard illumination with water depth adjusted to 15 cm, ensuring animals could neither touch the bottom with their hind limbs nor use tail support. Behavioral sessions were video-recorded using a lateral camera setup. Immobility time during the terminal 4-minute interval was quantified offline by a single blinded observer, defined as passive floating with only minor postural adjustments required to maintain buoyancy.

#### Social interaction test (SIT).

Experimental mice underwent the SIT comprising two sequential 2.5-minute phases. During the initial phase, subjects were placed in an open field arena (50 × 50 × 50 cm) containing an empty wire-mesh enclosure (Plexiglas, 10 × 6.5 × 42 cm). The subsequent phase introduced an unfamiliar CD1 mouse into the enclosure as a social target.

The interaction zone was defined as a 20 × 14 cm rectangular area extending 8.5 cm around the enclosure, with corner zones (9 × 9 cm) positioned at distal arena corners. All behaviors were recorded using the Noldus Observer XT 12.0 tracking system, with proximity within the interaction zone (≤8.5 cm from enclosure) constituting social interaction behavior.

#### Sucrose preference test (SPT).

Following individual housing, mice underwent a 4-day acclimation protocol: 2 days with dual water bottles followed by 2 days with dual 1% sucrose solutions. After 12-hour water deprivation, subjects received 24-hour access to one water bottle and one sucrose solution bottle, with lateral position reversal at the 12-hour midpoint. Sucrose preference ratio was calculated as [sucrose intake/(sucrose + water intake)] × 100%. Behavioral test data (FST, SIT and SPT) were available in S1 Table.

#### Peripheral blood collection and FFAs quantitative analysis.

For collection of human peripheral blood, samples were centrifuged at 3000 × g for 10 minutes at 4°C. Subsequently, 100 μL of the supernatant was carefully aspirated into a microcentrifuge tube while avoiding aspiration of cellular components. The aliquots were then stored in a designated human sample freezer (−80°C) pending GC–MS analysis. Further details regarding MC-MS were provided in the S1 File.

After completion of FST, SIT and SPT, 6 mice were randomly selected to collecte peripheral blood. Following ocular enucleation using curved forceps, exuding blood was carefully collected into 1.5-ml centrifuge tubes treated witht procoagulant and left at room temperature for 30–60 minutes to allow natural coagulation. Samples were then centrifuged at 3000 × g for 10 min at 4°C. The resulting supernatant, identified as serum, was stored at −80°C until further GC–MS analysis. The quantitative results of FFAs in alMDD and mice were seen in S2 Table.

#### Statistic analysis.

In all experiments, mice were randomly grouped, and data was analyzed using GraphPad Prism V10. Data distributions were evaluated using quantile-quantile (Q-Q) plots and Shapiro-Wilk tests prior to parametric modeling. * Asterisks indicate statistical significance: *p < 0.05 and **p < 0.01. According to the data distribution, we used unpaired t-test, Kruskal–Wallis test, two way-ANOVA, one way-ANOVA, Chi-square test and Spearman’s rank correlation. The differences in gender, alcohol intaking and smoking were analyzed using the chi-square test, while differences in age, years of education, HAMA-14 and HAMD-17 were analyzed using the Kruskal–Wallis test.

Z-score values were calculated by subtracting the mean of FFAs concentration in HC population from an individual raw value and then dividing this difference by the standard deviation from the HC population. We applied the S-score to compare the similarity of FFAs in CSDS mice and LPS mice with the items of FFAs in human peripheral blood. The S-score was derived using a formula where the mean z-score of alMDD was subtracted from an individual’s raw value, and the resulting difference was divided by the standard deviation of z-scores in the alMDD cohort.

## Results

### Participations characteristics

The study population comprised 204 participations, including 116 alMDD individuals and 88 healthy controls (HC). An overview of key demographic characteristics was summarized in Table 1. There was a significant difference in HAMA-14 and HAMD-17 scores with between alMDD and HC groups. Two-tail Chi-square test showed a significantly gender difference between alMDD and HC groups. However, comparison of smoking status, alcohol intaking, and the years of education showed no significant differences. No significant age differences in participations with alMDD and HC.

**Table 1:** Demographic and clinical characteristics.

Parameter	HC (mean±SD)	alMDD (mean±SD)	Test	*p-*value
n	88	116		
Gender(male/female)^a^	55/32	22/94	40.359	**
Age(years)^b^	14.89 ± 1.89	14.55 ± 17.01	1.526	0.217
Years of education^b^	9.01 ± 1.95	8.77 ± 1.74	0.587	0.443
Alcohol intaking^a^^,^c^^	73/14/1	91/19/6	2.509	0.285
Smoking^a^^,^d^^	87/0/1	110/1/5	8.279	0.407
HAMA-14^b^	0.93 ± 1.30	24.16 ± 8.13	151.355	**
HAMD-17^^b^^	1.23 ± 2.20	22.17 ± 5.94	151.017	**

Continuous variables were presented as the mean ± SD (standard deviation). * p < 0.05, ** p < 0.01. alMDD adolescent patient with major depressive disorder, HC health control, HAMD-14 14-item Hamilton depression rating scale, HAMA-17 17-item Hamilton anxiety rating scale.

^a^Analyzed by Chi-square test.

^b^Analyzed by Kruskal–Wallis test.

^c^Alcohol intaking, abstinence (no alcohol use)/social drinking (occasional alcohol intake in social settings)/regular alcohol use (systematic alcohol consumption ≥3 times/week)

^d^Smoking, never smoking (Lifetime cumulative smoking <5 packs [1 pack = 20 cigarettes])/former smoking (Cumulative smoking ≥5 packs with cessation sustained for ≥3 months)/current smoking (daily consumption >1 cigarette/day with lifetime cumulative smoking ≥5 packs)

### The peripheral blood FFAs levels were generally decreased in alMDD

**Fig 1:**
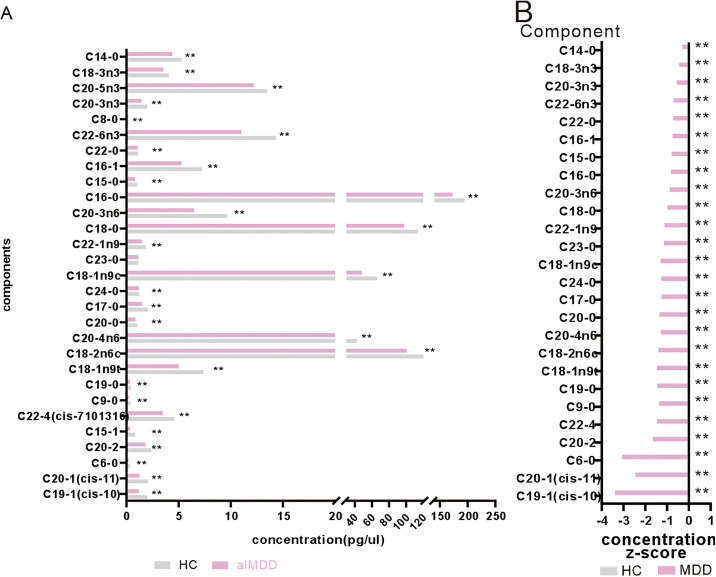
Schematic changes of FFAs in alMDD and HC. **(A)** Quantitative profiles of FFAs in alMDD and HC. **(B)** Standardized distribution of FFAs following z-score transformation. Mann Whitney test. The data are presented as the mean ± SEM; *p < 0.05.

To investigate the specific changes of FFAs between alMDD and HC individuals, GC-MS was employed to quantify of FFAs levels in peripheral blood samples from participants (Table 1 in S2 Table). Following normality assessment, FFAs demonstrating non-normal distributions underwent nonparametric test. To address potential confounding effects, a general linear model (GLM) was employed to adjust for sex-related variations in FFAs levels (Tables 1 and 2 in S3 Table). For each metabolite, a GML was fitted with alMDD as the dependent variable while adjusting for FFAs. The characteristics of FFAs concentrations are reported in Tables 1 and 2 in S3 Table. The characteristics of FFAs in participationts exhibited statistically significant intergroup differences in circulating FFAs profiling (Fig 1A and Table 2 in S3 Table). We found that alMDD individuals exhibited a significant downregulation of peripheral blood FFAs compared to HC.

To enable the direct comparison of FFAs levels between alMDD and HC, the concentrations of FFAs from both groups were standardized using z-score transformation (Table 1 in S3 Table). Compared to HC, the concentrations of FFAs in alMDD individuals showed an overall down-regulation trend (Fig 1B and Table 2 in S3 Table). Fig 1A showed the kinds and distributions of FFAs in human. The above evidence showed that the concentrations of the peripheral blood FFAs were generally reduced in alMDD.

### Expression level changes of FFAs in the peripheral blood in CSDS mice and LPS mice

**Fig 2:**
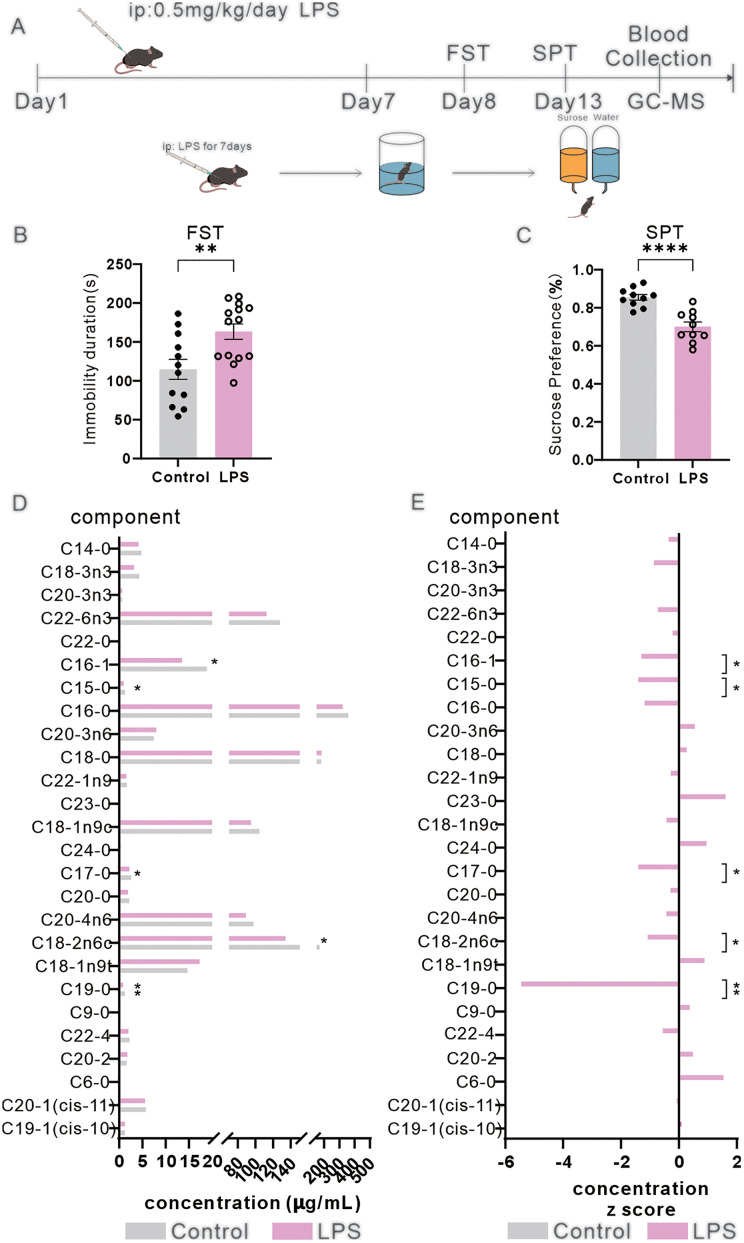
Partial FFAs exhibit downregulation in LPS mice. **(A)** Schematic of LPS-induced depressive modeling and fatty acid detection. B-C Depressive-like behavioral assessments (t-test, n = 10). **(B)** Immobility time in the forced swim test (FST). **(C)** Sucrose preference test (SPT) (t-test). **(D)** Concentrations of FFAs in the peripheral blood of LPS mice. **(E)** Z-score standardized concentrations of FFAs in LPS mice (t-test). The data are presented as the mean ± SEM; *p < 0.05, **p < 0.01.

**Fig 3:**
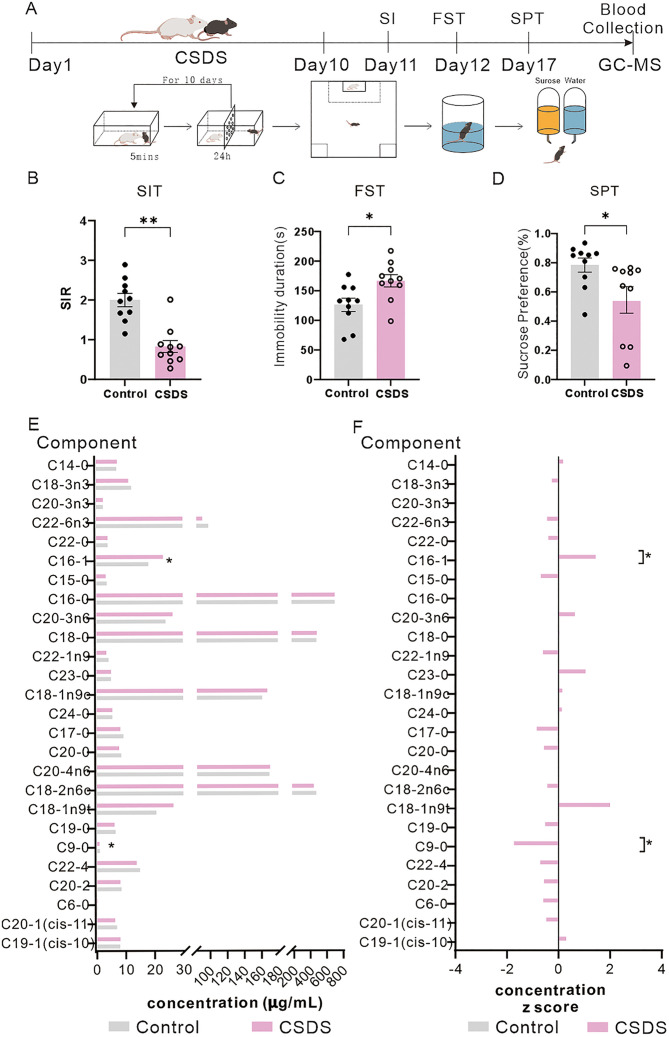
Partial FFAs exhibit downregulation in CSDS mice. **(A)** Schematic of CSDS and fatty acid detection. B-C Depressive-like behavioral assessments (t-test, n = 10). **(B)** Social interaction (SI) test. **(C)** Immobility time in the forced swim test (FST). **(D)** Sucrose preference test (SPT). **(F)** Z-score standardized concentrations of FFAs in LPS mice (independent samples t-test). The data are presented as the mean ± SEM; *p < 0.05, **p < 0.01.

To compare alterations of FFAs levels in LPS mice and CSDS mice separately, we established both LPS and CSDS paradigms (Figs 2A and 3A). Peripheral blood was collected from LPS-treated and CSDS-exposed mice for quantitative FFAs profiling. Following successful depression modeling, mice exhibited depressive-like behaviors, including reduced social interaction in CSDS mice, and both of two paradigms showed the prolonged immobility durations in the FST, and diminished sucrose preference (Figs 2A–2D and 3A–3C–3C).

Although systemic FFAs downregulation was observed in both model post-induction, distinct patterns of individual FFAs species were identified between LPS and CSDS mice. Summarized statistic of FFAs in the above two models were reported in Tables 3–6 in S3 Table. Among the quantified 26 items of FFAs, LPS mice showed significant reductions in 5 components of FFAs, including nonadecylic acid (C19:0), trans-9-octadecenoic acid (C18:2n6c), heptadecanoic acid (C17:0), pentadecanoic acid (C15:0), and cis-9-palmitoleic acid (C16:1) (Fig 2D and 2E). CSDS mice exhibited decreased levels of 2 items of FFAs: nonanoic acid (C9:0) and C16:1 (Fig 3E and 3F). These results demonstrated that FFAs levels were differentially suppressed in the two depressive models, while LPS mice showing a broader spectrum and higher items of FFAs reductions (Tables 3–6 in S3 Table). Notably, C16:1 level was consistently downregulated in both two models, suggesting a shared metabolic perturbation associated with depression-like phenotypes. Quantitative lipidomics revealed divergent FFAs signatures in LPS mice versus CSDS mice, suggesting inflammation-driven and stress-induced lipid metabolic pathways contribute differentially to depression-like sub-phenotypes.

### The FFAs levels in LPS mice more closely recapitulate to adolescent patients with MDD versus CSDS mice

**Fig 4:**
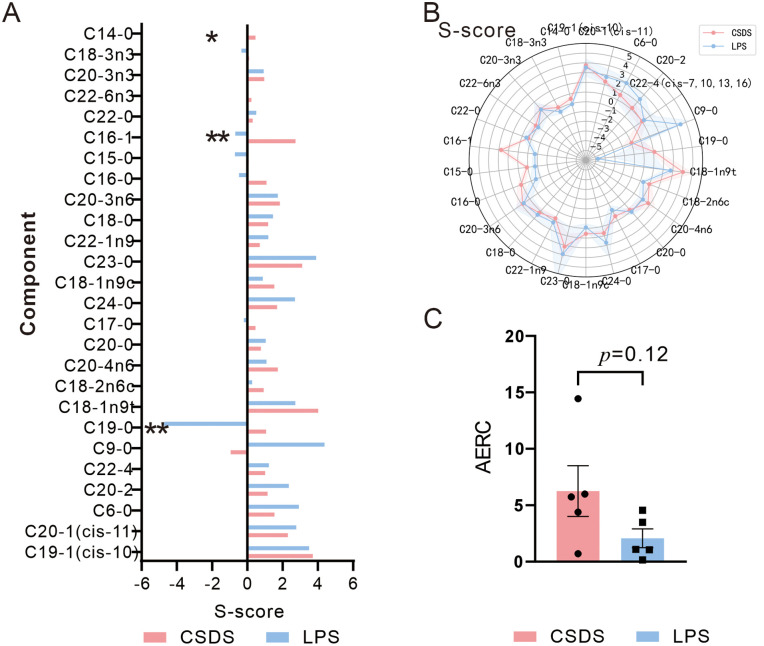
Comparative analysis of FFAs Between LPS mice and CSDS mice reveals closer alignment of LPS-induced FFAs profiles with alMDD. **(A)** S-score comparison of FFAs levels between LPS and CSDS mice (two-sample t-test, n = 6). **(B)** Radar plot illustrating FFAs profiles in LPS mice and CSDS mice. (C) s-score-derived AERC profile of FFAs in LPS mice and CSDS mice. AERC Area enclosed by the radar chart for FFAs in mice. The data are presented as the mean ± SEM; *p < 0.05, **p < 0.01.

To enable comparative analysis of FFAs levels between LPS mice, CSDS mice, and human with alMDD, we applied the s-score metric meaning that the more s-score close to zore, the model better stimulating FFAs levels in alMDD, as detailed in Table 7 in S3 Table. The s-score reflects an individual’s deviation from population norms, with values approaching zero indicating closer alignment with the population average, while higher absolute values denote greater divergence from normative ranges. CSDS mice exhibited s-score indicating significant alignment of hexanoic acid (C6:0), C9:0, and C19:0 with the FFAs profile of alMDD, and LPS mice demonstrated closer approximation to alMDD FFAs levels in C16:1 and myristic acid (C14:0) (Fig 4A). To further visualized the overall distributional disparities in FFAs dysregulation between CSDS mice and LPS mice murine models relative to human profiles, we analyzed the polygon areas of radar plots constructed using the s-score metric quantifying directional similarity in FFAs alterations. By calculating the area enclosed by the radar chart (AERC) for FFAs in each mouse, we found that the overall peripheral FFAs levels in CSDS individuals were higher than those in LPS individuals (Fig 4C and Table 8 in S3 Table). Notably, LPS mice displayed a greater number of differentially regulated FFAs compared to CSDS mice, with these FFAs also showing systemic downregulation alMDD cohorts. The mean AERC of LPS mice was 1.854, although this value deviates from zero, the overall trend indicates that their FFAs levels better simulate those of alMDD.

Our study demonstrates that the peripheral blood FFAs levels in alMDD are downregulated. However, in depressive-like animal models, the FFAs profiles of CSDS and LPS mice differ. In CSDS mice, peripheral blood levels of C19:0, C18:2n6c, C17:0, C15:0, and C16:1 were decreased. while the peripheral blood levels of C9:0 and C16:1 also showed reductions in PLS mice. Importantly, we noted that the LPS mice more closely recapitulates the FFAs dysregulation observed in alMDD than the CSDS mice.

## Disscussion

This study based on previous study that the FFAs were associated with multiple disease and existed down-regulation changes in alMDD [25]. Our study provide evidence to deconstructs that the LPS mice performs closer to alMDD in the FFAs profiling, meaning that LPS mice were much appropriate to build depression-like behavior model particularly for FFAs components analysis. Employing GC-MS quantifying the peripheral blood FFAs, we identify that the concentrations of FFAs get decreased in total in alMDD individuals. Moreover, the levels of FFAs in the peripheral blood show a downregulation trend particularly both in CSDS mice and LPS mice, but there are higher items of FFAs get decreased in CSDS mice generally without significant difference. Meanwhile, when it comes to study C6-0 and C9-0 and C19-0, the CSDS mice are worthy to choose for building the depression-like behavior model while the LPS mice are more available for further study inC16-1 and C14-0 profiling in simulating the peripheral blood changes of FFAs in alMDD. Our finding offer insight for choosing LPS induced depression-like model targets for animal depression research in peripheral blood FFAs assaying.

Our study demonstrated the crucial fluctuation of concrete components FFAs in LPS mice, extending the importance of choosing specific model for FFAs analysis to the field of depression. As the sample size limitation of CSDS mice and LPS mice, there may exist heterogeneity in the levels of FFAs in mice, but the comparison between groups can be guaranteed by converting to the z-score and S-score, indicating that our results are reliable. Although studies have been made in merging FFAs therapy and potential biomarker at the strategy in MDD patients, in preclinical animal study its underlying molecular mechanisms at the cellular level remain largely unknown [28–30]. The LPS-induced depression-like mice remain the fluctuation of concentrations principally in line with MDD individuals. Base on the quantitative method, our finding determined the peripheral blood FFAs levels in LPS mice and CSDS mice. Although significant downregulation of C19:0, C18:2n6c, C15:0, and C16:1 was identified in LPS-treated mice, and C9:0 and C16:1 in CSDS-exposed mice, none of these FFAs has been linked to depression for a specific pathological mechanism in previous evidence. Future investigations are warranted to elucidate their roles in depressive pathophysiology, with potential implications for advancing diagnostic biomarkers, therapeutic interventions, and preventive strategies targeting lipid metabolic dysregulation in depression.

The role of inflammation in MDD has been increasingly recognized [11]. In animal models, studies on both CSDS mice and LPS mice suggest that inflammation plays a critical role in mediating the development of depressive-like behaviors [31–35]. Given the differential expression of peripheral blood FFAs between CSDS and LPS mice, we hypothesize that certain FFAs may be associated with stress-related pathways, while others are linked to inflammatory responses. Our study revealed that the levels of C6:0 (hexanoic acid), C9:0 (nonanoic acid), and C19:0 (nonadecanoic acid) in CSDS mice and C14-0, C16-1 in LPS mice closely resembled those observed in humans. Furthermore, previous studies have demonstrated that Cistanche tubulosa extract (CTE) modulates C6:0 levels in stressed mice, and that C6:0 exerts protective effects against oxidative stress in erythrocytes [36,37]. Studies have also reported that nonanoic acid (C9:0) exacerbates allergic inflammation in mice [38]. However, its role in stress-related responses and inflammatory pathways during depression remains unclear. Additionally, nonadecanoic acid (C19:0) has been linked to oxidative stress; however, its role in major depressive disorder (MDD) remains unexplored [39]. While myristic acid (C14:0) has been implicated in both stress-related pathways and inflammatory responses, accumulating evidence suggests a more prominent association with inflammatory responses [40–42]. The C16:1 (palmitoleic acid) levels were significantly reduced in both depressive models, but the reduction in LPS mice closely mirrored levels observed in alMDD, suggesting a stronger association with inflammatory pathways. In summary, C6:0 (hexanoic acid), C9:0 (nonanoic acid), and C19:0 (nonadecanoic acid) may be more strongly associated with stress-related pathways, while C14:0 (myristic acid) and C16:1 (palmitoleic acid) appear to correlate predominantly with inflammatory responses. However, further investigation is required to elucidate their specific roles in the pathophysiology of MDD.

Several limitations should be acknowledged in this study. A significant gender difference is noted between alMDD and HC, with a higher proportion of females in the alMDD cohort. This finding is consistent with the well-established epidemiological fact that MDD is more prevalent in women [43,44]. For instance, one study of 2,541 individuals reported that approximately two-thirds of the participants were female, and that women with MDD exhibited significantly greater symptom severity than men [45]. This included a higher prevalence of comorbid anxiety disorders, bulimia, and somatoform disorders, as well as a greater number of past suicide attempts. Some scholars postulate that this disparity may be attributable to genetic factors that confer a heightened susceptibility to depression in females [46]. Consequently, considerable research efforts have been directed toward elucidating the mechanisms underlying these observed sex differences in depressive disorders; however, no definitive mechanisms have been established to date. The modest sample size of animal cohorts may have reduced statistical power to detect subtle differences in FFAs profiles. While our investigation was restricted to male mice, precluding an assessment of potential sex-specific variations in FFAs dynamics. FFAs are influenced by confounding variables such as diet, geographic factors, and physiological states, which likely contributed to incomplete alignment between murine FFA patterns and those observed in adolescents with MDD. Our study focused exclusively on LPS mice and CSDS mice, leaving other established depression paradigms unexplored. With sufficient sample size, we found that the peripheral blood FFAs were downregulated in alMDD individuals, with most detected species at low concentrations. The low concentrations of FFAs may reveal the distinct FFAs pathological mechanism dynamics in depression. Acting of metabolite, the research for FFAs provided plenty of evidences for further study in preclinical trail [47–50]. However, concrete FFAs have also been implecated in depression, further researches are warranted to clarify whether these FFAs contain and its mechanism in depression. And the selection of an appropriate depressive model is critical for investigating FFAs.

## Supporting information

S1 Table Characteristics of behavioral test in LPS and CSDS mice. Table 1 the behavioral data in LPS mice. Table 2 the behavioral data in CSDS mice. *p*-value was caculated by t-test.
(XLSX)

S1 File Supplementary method of GC-MS Detailed information for GC-MS.
(PDF)

S2 Table Concentrationt characteristics of FFAs in human individuals, LPS mice and CSDS mice. Table 1 quantitative analysis of FFAs in human beings (μg/mL). Table 2 quantitative analysis of FFAs in LPS mice (μg/mL). Table 3 quantitative analysis of FFAs in CSDS mice (μg/mL).
(XLSX)

S3 Table Characteristics of FFAs in LPS mice and CSDS mice by z-score and S-score. Table 1 z-score characteristics of FFAs in human individuals. Table 2 characteristics of FFAs in human peripheral blood. The general linear model was adjusted by including covariates with the following parameter estimates: years of education = 8.87, age = 14.70, and gender = 1.62. Mann Whitney test. The data are presented as the mean ± SEM. Table 3 the concentrationt characteristics of FFAs in CSDS mice. Table 4 characteristics of FFAs in CSDS mice peripheral blood. The data are presented as the mean ± SEM. Table 5 the concentrationt characteristics of FFAs in LPS mice. Table 6 characteristics of FFAs in LPS mice peripheral blood. The data are presented as the mean ± SEM. Table 7 characteristics of S-score in mice. The data are presented as the mean ± SEM. Table 8 S-score-based AERC profile of FFAs in mice. AERC area enclosed by the radar chart.
(XLSX)
