# Supplementary material for: Lipopolysaccharide induced mouse depression model can better simulate changes in peripheral blood FFAs in alMDD
Source: PLoS One. 2026 Jan 23;21(1):e0340967. doi: 10.1371/journal.pone.0340967 (PMC12829858; doi:10.1371/journal.pone.0340967)
Supplement: S1 File — Detailed information for GC-MS. (PDF) [file pone.0340967.s002.pdf]

Supplementary Method of GC-MS

1 Gas chromatography-mass spectrometry

1.1 Reagents and instruments

Instruments	Type	Brand
GC-MS/MS	7890B-7000D	Agilent
GC-MS	8890-5977B	Agilent
Centrifuge	5424R	Eppendorf
Electronic balance	AS 60/220.R2	RADWAG
Ball crusher	MM400	Retsch
SpeedVac	CentriVap	LABCONCO
Multi-tube Vortexer	MIX-200	Jingxin
Mini mixer	MIX-25P	MIULAB
Ultrasonic cleaner	KQ5200E	Shumei
Dry box	DHG-9055A	Heheng

Reagents	grade	Brand
methanol	HPLC	Merck
methyl tert-butyl ether	HPLC	Merck
phosphoric acid	HPLC	Sigma-Aldrich
hexane	HPLC	CNW
15% boron trifluoride methanol	HPLC	RHAWN
sodium chloride	HPLC	Sigma-Aldrich

1.2 Sample Preprocessing

(1) Retrieve frozen specimens from -80 ℃ storage and equilibrate on ice for 30 minutes. Maintain all subsequent procedures at 0-4 ℃ conditions.

(2) Following complete thawing, vortex-mix samples vigorously for 10s using a benchtop vortexer. Precisely aliquot 50 μL of homogenized sample into a pre-chilled 1.5 mL polypropylene microcentrifuge tube.

(3) Sequentially add three extraction solvents:

- 150 μL HPLC-grade methanol
- 200 μL methyl tert-butyl ether (MTBE)
- 50 μL phosphoric acid solution (36%, v/v)

(4) Perform phase separation through:

- Intensive vortex mixing (3 min at maximum speed)
- Centrifugation (5 min at 12,000 ×g, 4 °C)

(5) Carefully collect 200 μL of the upper organic phase and concentrate to complete dryness using a nitrogen evaporator (40 °C bath temperature).

(6) Derivatize residues with 300 μL BF_3-methanol complex (15% w/v) through:

- Immediate vortex reconstitution (3 min)
- Thermal activation (30 min incubation at 60 °C)

(7) Cool derivatives to ambient temperature (22±2 °C), then add:

- 500 μL n-hexane (chromatographic grade)
- 200 μL NaCl-saturated aqueous solution

(8) Finalize sample preparation by:

- Phase mixing (3 min vortex)
- Low-temperature centrifugation (2 min at 12,000 ×g, 4 °C)
- Precise collection of 100 μL n-hexane phase for instrumental analysis

1.3 Collection conditions for chromatography and mass spectrometry

**Mass spectrometry conditions**	**parameter**
Injection volume	1 μL
Front Inlet Mode	splitless
Carrier Gas	Helium
Column	DB-5MS (30 m x 0.25 mm x 0.25 μm)
Column Flow	1 mL/min
Oven Temperature Ramp	40°C（2min）, raised to 200°C at a rate of 30°C /min，200°C（1min），raised to 240°C at a rate of 10°C /min ，240°C（1min），raised to 285°C at a rate of 5°C /min，285°C（3min）
Transfer Line Temperature	240 °C
Ion Source Temperature	230 °C
Quad Temperature	150 °C
Electron Energy	70 eV

1.4 Standard curve

The chromatographic peak intensity data were acquired by preparing a gradient series of standard solutions at concentrations of 0.01, 0.02, 0.05, 0.1, 0.2, 0.5, 1, 2, 5, 10, 20, and 50 μg/mL. Calibration curves were established by plotting the concentration ratio (analyte/internal standard) on the abscissa against the corresponding peak area ratio (analyte/internal standard) on the ordinate. The linear regression equations and their correlation coefficients for all target analytes are systematically tabulated below.

Index	Class	RT	Equation
C6-0	lipid	4.53305	y = 6073740.872772 x - 12296.624527
C8-0	lipid	5.75455	y = 71007.790324 x - 243.291253
C9-0	lipid	6.282966667	y = 113327.885345 x - 521.390473
C10-0	lipid	6.769266667	y = 222124.612282 x - 603.541824
C11-1(cis-10)	lipid	7.186666667	y = 1802245.719117 x - 15895.523979
C11-0	lipid	7.222083333	y = 6809977.458088 x - 22519.218677
C12-0	lipid	7.668333333	y = 454777.734588 x - 2098.755988
C13-0	lipid	8.17835	y = 585981.219774 x - 3624.177808
C14-1	lipid	8.709616667	y = 155065.468575 x - 851.984799
C14-0	lipid	8.78045	y = 749804.104962 x - 5451.948442
C15-1	lipid	9.389633333	y = 154820.911250 x - 1638.393213
C15-0	lipid	9.460483333	y = 899502.742621 x - 8021.808169

C16-1	lipid	10.06258333	y = 189326.008367 x - 7305.296413
C16-1T	lipid	10.09091667	y = 148054.867715 x + 4191.735718
C16-0	lipid	10.21133333	y = 937906.111459 x - 6520.996134
C17-1	lipid	10.84176667	y = 150486.689015 x - 980.007608
C17-1T	lipid	10.88426667	y = 56759.243360 x - 1149.262662
C17-0	lipid	11.00468333	y = 1075035.370213 x - 9772.593298
C18-3n6	lipid	11.4297	y = 167100.056522 x - 7498.829291
C18-2n6c	lipid	11.56428333	y = 397129.392390 x - 16284.890243
C18-3n3	lipid	11.61386667	y = 157080.138478 x - 1628.742310
C18-1n9c	lipid	11.62095	y = 185039.832507 x + 522.138885
C18-2n6t	lipid	11.62095	y = 500269.410365 x - 5093.524371
C18-1n9t	lipid	11.66345	y = 194687.836180 x - 1948.967015
C18-1(trans-11)	lipid	11.691	y = 126940.892326 x - 1445.492321
C18-0	lipid	11.8193	y = 1302644.938190 x - 13297.353670
C19-1(cis-10)	lipid	12.43556667	y = 108814.897351 x - 911.317305
C19-0	lipid	12.64806667	y = 930498.025154 x - 8897.362329
C16-2	lipid	12.87475	y = 1052540.340015 x - 48393.408979
C20-4n6	lipid	12.9385	y = 16660.203201 x - 444.878681
C20-5n3	lipid	13.00225	y = 56964.225218 x - 2523.411469
C20-3n6	lipid	13.1085	y = 172661.996101 x - 3549.842270
C20-2	lipid	13.29975	y = 372145.204603 x - 18297.734481
C20-1(cis-11)	lipid	13.35641667	y = 123253.276800 x - 1181.170409
C20-3n3	lipid	13.37058333	y = 186337.649357 x - 8684.983403
C20-1T	lipid	13.42016667	y = 122081.578412 x - 2561.287066
C20-0	lipid	13.61143333	y = 1430721.658566 x - 31957.977051
C21-0	lipid	14.69521667	y = 1433654.345191 x - 66373.445861
C22-6n3	lipid	14.93605	y = 2849204.649086 x - 180367.054710
C22-4(cis-7,10,13,16)	lipid	15.02813333	y = 23559.557821 x - 2475.197768
C22-5(cis-7,10,13,16,19)	lipid	15.10605	y = 5903.695404 x - 446.047268
C22-2	lipid	15.49565	y = 331312.289204 x - 18512.949651
C22-1n9	lipid	15.55231667	y = 111613.878958 x - 5658.038310
C22-1T	lipid	15.62315	y = 95198.595523 x - 3974.038582
C22-0	lipid	15.84275	y = 1557849.692974 x - 76615.023984
C23-0	lipid	17.03986667	y = 1452225.422918 x - 81034.460428
C24-1	lipid	17.97488333	y = 75941.276539 x - 8173.253768
C24-0	lipid	18.2724	y = 1434342.741468 x - 79859.299989

1.5 sample concentration calculation

Free Fatty acid (FFAs) quantification was performed using the following analytical workflow:

1. Chromatographic peak area integrals from experimental samples were interpolated into the established standard curve linear regression model (y = ax + b) to determine preliminary concentration values.

2. The absolute FFAs content in original specimens was calculated using the normalized formula:

$$\text{FFAs Concentration } (\mu\text{g/mL}) = \frac{c \times V3 \times V1}{1000 \times V2 \times V0}$$

Where:

c: Analyte concentration derived from the calibration curve (μg/mL)

V0: Original sample volume taken for processing (μL)

V1: Volume of primary extraction solvent (μL)

V2: Supernatant aliquot volume collected after phase separation (μL)

V3: Volume of derivatized residue reconstitution solution (μL)
